# Seasonal Hair Glucocorticoid Fluctuations in Wild Mice (*Phyllotis darwini*) within a Semi-Arid Landscape in North-Central Chile

**DOI:** 10.3390/ani14091260

**Published:** 2024-04-23

**Authors:** Joseline Veloso-Frías, Mauricio Soto-Gamboa, Gabriela Mastromonaco, Gerardo Acosta-Jamett

**Affiliations:** 1 Institute of Preventive Veterinary Medicine, Austral University of Chile, Valdivia 5090000, Chile; josy.velosoo@gmail.com; 2 Institute of Environmental and Evolutionary Sciences, Austral University of Chile, Valdivia 5090000, Chile; mrsoto@uach.cl; 3 Reproductive Physiology, Toronto Zoo, Toronto, ON M1B 5K7, Canada; gmastromonaco@torontozoo.ca; 4 Center for Surveillance and Evolution of Infectious Diseases (CSEID), Austral University of Chile, Valdivia 5090000, Chile

**Keywords:** anthropization, drylands, glucocorticoids, hair corticosterone, *Phyllotis darwini*, rodents, stress response

## Abstract

**Simple Summary:**

In drylands, mammals face inhospitable environmental conditions exacerbated by climate change. Currently, these environments are extensively intervened by human activity, and it is unknown whether this could increase the energy demands experienced by individuals. Corticosterone is a hormone that increases in response to the energy needs of individuals, which varies with health and developmental state. We assessed how human activity, individual characteristics, and seasonality influence corticosterone accumulated in the hair of the Darwin’s leaf-eared mouse (*Phyllotis darwini*) inhabiting two areas with contrasting human impact in a semi-arid ecosystem of northern Chile. We collected 199 hair samples and recorded sex, body condition, and ectoparasite load, variables that could influence corticosterone concentrations. We found no differences in accumulated hair corticosterone between anthropized areas and areas protected from human disturbance; however, higher concentrations were recorded in females, and males showed seasonal fluctuations. The findings could suggest that additional factors or their interplay exert a more substantial influence on corticosterone concentration than human disturbance. Additionally, the influence of sex and seasonality on hair corticosterone concentration in *P. darwini* makes it necessary to include these variables in future assessments of this species.

**Abstract:**

Mammals in drylands face environmental challenges exacerbated by climate change. Currently, human activity significantly impacts these environments, and its effects on the energy demands experienced by individuals have not yet been determined. Energy demand in organisms is managed through elevations in glucocorticoid levels, which also vary with developmental and health states. Here, we assessed how anthropization, individual characteristics, and seasonality influence hair glucocorticoid concentration in the Darwin’s leaf-eared mouse (*Phyllotis darwini*) inhabiting two areas with contrasting anthropogenic intervention in a semi-arid ecosystem of northern Chile. Hair samples were collected (n = 199) to quantify hair corticosterone concentration (HCC) using enzyme immunoassays; additionally, sex, body condition, and ectoparasite load were recorded. There were no differences in HCC between anthropized areas and areas protected from human disturbance; however, higher concentrations were recorded in females, and seasonal fluctuations were experienced by males. The results indicate that animals inhabiting semi-arid ecosystems are differentially stressed depending on their sex. Additionally, sex and season have a greater impact on corticosterone concentration than anthropogenic perturbation, possibly including temporal factors, precipitation, and primary production. The influence of sex and seasonality on HCC in *P. darwini* make it necessary to include these variables in future stress assessments of this species.

## 1. Introduction

Arid and semi-arid environments present environmental challenges for mammals, which have been exacerbated under the scenario of climate change [1]. These challenges include intense solar radiation, high temperatures, and scarce precipitation, which constrain primary production, thereby affecting the trophic chain and potentially intensifying competition for resources [1,2,3]. Under adverse environmental conditions, such as those mentioned, animals experience an increase in energy demand [4], regulated by the hypothalamic–pituitary–adrenal (HPA) axis, resulting in elevated blood levels of glucocorticoids (GC) [4,5]. These hormones stimulate physiological responses and behaviors to maintain homeostasis and cope with stressful stimuli [4,6]. They promote gluconeogenesis and cognition while downregulating less essential functions in response to stressors, such as reproduction, immune function, digestion, and growth [6,7,8].

Additionally, individuals inhabiting drylands must cope with energy demands inherent to their development and health status, factors that impact their GC levels [4,9,10]. For instance, multiple studies reveal differences in GC concentrations between sexes [9], associated with dissimilarities in energy costs related to reproduction and social interactions [9,11,12,13,14] and the differentiated stimulation of sexual hormones in the HPA axis [15]. Moreover, variation in hormone levels has been observed across different reproductive states [9,15], with increases during periods of higher energy demand [9,16,17], as well as in individuals with poor body condition [18,19,20]. Regarding health status, infectious and parasitic diseases incur energy costs by mounting and sustaining the immune response [5,21], consuming host nutrients [22], and altering behavior, thereby negatively impacting intake and rest [23,24,25]. Consequently, on multiple occasions, these states have been associated with increases in GC concentrations [9,26,27,28].

Currently, drylands are recognized as sensitive areas to human activity [29], with a significant portion of their surface being impacted by it [30]. This impact is often associated with agriculture, grazing, resource extraction, and urbanization [30,31]. Anthropization can alter habitats and extend its effects over time by modifying the availability or quality of resources, subjecting individuals to new stressors [5]. Despite this, it has not been precisely determined how anthropogenic activity could impact the energy demand and physiological stress response (level of GC) experienced by individuals inhabiting these areas. However, increased CG has been associated with more fragmented habitats in small rodents living in the Atlantic Forest (Paraguay), suggesting that anthropic activity could be a critical stressor for rodents in natural areas [32].

While an increase in GC may be beneficial for the individual in the short term [5], prolonged elevation, and consequently, sustained suppression of biological functions, predispose to reproductive problems [5,7], poor body condition [4], and immunosuppression [5,33,34], thereby threatening survival [35,36]. Given the potential negative effects of chronic GC elevation in wild populations, it is crucial to determine the factors influencing these hormone concentrations. Measurements of hair glucocorticoid concentrations (HCCs) have recently been used to demonstrate response to chronic stressors, as they reflect accumulated blood GC concentration over extended periods [37], making them a valuable biomarker for assessing the impact of anthropization on stress physiology [32,38,39]. Considering the aforementioned and the prolonged energy constraints faced by fauna in drylands, we conducted seasonal monitoring of HCC in two rodent populations inhabiting areas with different degrees of anthropogenic activity in a semi-arid zone of northern Chile. We chose the Darwin’s leaf-eared mouse (*Phyllotis darwini*) as the study species, one of the most abundant micromammals in the area [40]. We recorded and assessed whether variations in GC concentrations are associated with locality, seasonality, sex, body condition, and ectoparasitic load.

The aim of our study was to assess whether environmental and intrinsic factors contribute to physiological stress in the population of *P. darwini* inhabiting a semi-arid landscape in north-central Chile. We evaluated these objectives under the hypothesis that environmentally challenging conditions and energetically demanding life states induce increases in GC concentrations, predicting higher concentrations in rodents inhabiting anthropized sites during food-restricted seasons and in energetically demanding developmental or health states.

## 2. Materials and Methods

### 2.1. Study Area

**Figure 1:**
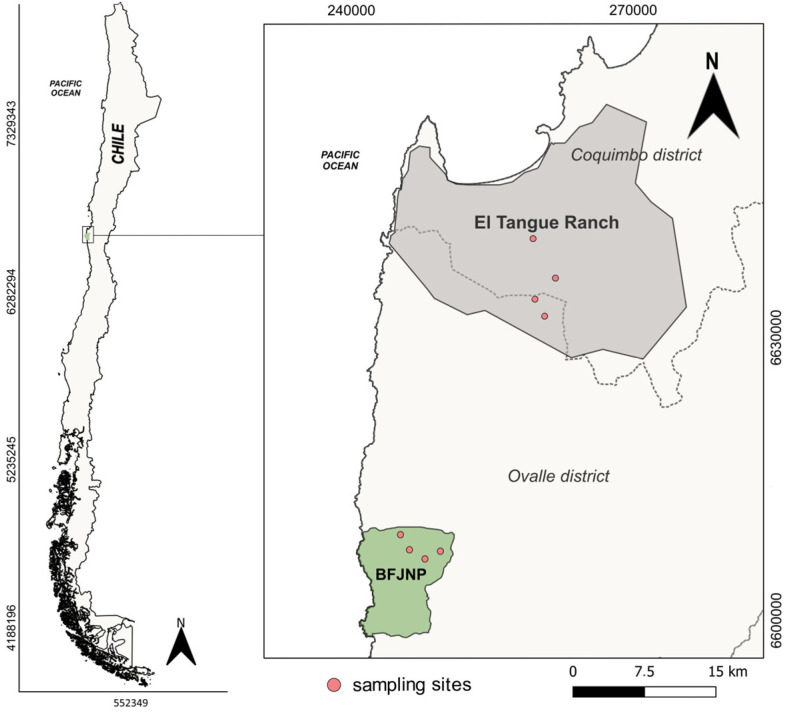
Localities and capture sites of Darwin’s leaf-eared mouse (*Phyllotis darwini*) in the coastal area of the Coquimbo region (UTM projection. Datum WGS84, Zone 19J). The Bosque de Fray Jorge National Park is shown in green and locality of Hacienda el Tangue in gray.

The study was conducted on the coast of the Coquimbo region in north-central Chile (71°12′ to 71°40′ W, 29°58′ to 30°39′ S) (Figure 1). This is a semi-arid Mediterranean zone with an average annual precipitation of around 100 mm, concentrated in short periods mainly during winter (June to September), with warm and dry summer months (December to March). The temperature ranges seasonally, with minimum and maximum ranging from 5 to 25 °C in autumn, 0 to 15 °C in winter, 5 to 20 °C in spring, and 10 to 30 °C in summer [41,42].

Two localities with different degrees of anthropogenic intervention were included in the study: the Bosque Fray Jorge National Park (BFJNP) and the rural locality of Hacienda El Tangue. BFJNP is a World Biosphere Reserve of 9959 ha [43]. Its dominant vegetation includes thorn scrub and succulents. This zone is safeguarded from anthropogenic activities [44], with a reported high level of anthropization for only 1.15% of its surface area, in contrast to 22% for the surrounding area [45]. 

Hacienda El Tangue is a rural locality located 10 km south of Tongoy town and 37 km north of the PNBFJ. The area consists of pastures, agricultural fields, and a mix of exotic shrubs and pioneer native vegetation. Its main productive activities are agriculture and traditional subsistence farming of sheep and goats. In recent years, there has been significant population growth in the area, accompanied by an increase in housing. Currently, El Tangue is connected to the country’s main land route (Pan-American Highway) via a paved road (Route D-510) that traverses the zone. 

### 2.2. Study Design

**Figure 2:**
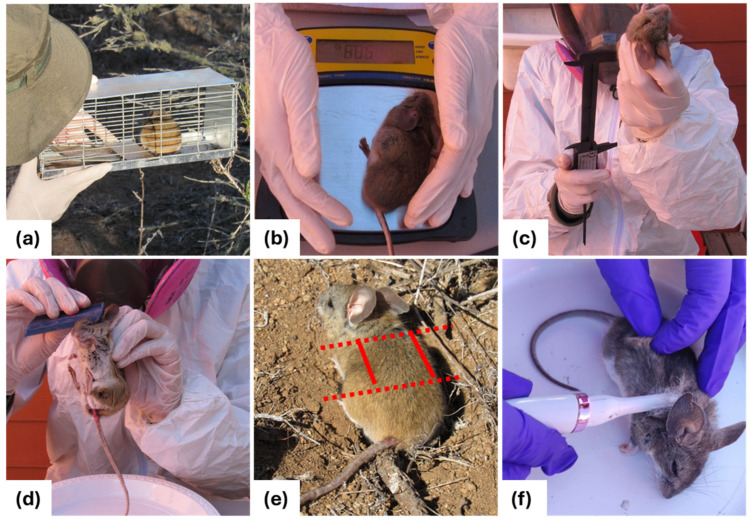
Field sampling sequence included the followimg: (**a**) capture of rodents using Sherman traps; (**b**) weighing of rodents on a digital scale; (**c**) measurement of morphometric parameters; (**d**) extraction of ectoparasites using a fine-tooth comb; (**e**) selected area for shaving the hair, with dashed lines located at the level of the scapulae and at the beginning of the lumbar area; (**f**) shaving of the hair with an electric trimmer.

Rodent captures were carried out between spring 2021 and summer 2023 in the BFJNP and Tangue. The target species was *P. darwini*, as it has been documented as dominant in both areas [40]. Four sampling sites were established in each location, and samples were consecutively taken for four nights in each sampling season. At each sampling site, 150 × 135 m grids with 200 Sherman traps were set. Each grid consisted of 10 main rows of 11 Sherman traps distributed equally spaced 15 m apart from each other. Additionally, 90 traps were set at the center of each grid cell formed by the main rows. Due to the nocturnal activity of *P. darwini*, the traps were baited with oat flakes and activated daily at dusk. Each trap was checked the following morning (Figure 2a), removing those with captures and deactivating the rest.

### 2.3. Morphometric Data and Sample Collection

Each captured rodent was weighed using a digital scale (Pesamatic Newton Series^®^, Model EJ1500, A&D Weighing, San Jose, CA, USA; ±0.1 g SD) (Figure 2b) and underwent sedation using a combination of Ketamine (0.044 mg/g) and Xylazine (0.006 mg/g) administered intramuscularly for handling [46]. Total length (from head to the base of the tail) and anogenital distance were measured with a digital caliper (Uberman^®^, precision 0.01 mm) (Figure 2c). Then, ectoparasites were collected for five minutes using fine tweezers and a fine-tooth comb (Figure 2d). Finally, a hair sample was obtained using an electric shaver by shaving a 2 cm^2^ area close to the skin along the dorsal midline between the shoulder blades and the anterior limit of the lumbar area (Figure 2e). This region was chosen due to studies showing potential variations in corticosterone measurements depending on the anatomical region of the obtained hair and to reduce urine contamination [32,47,48]. After sample collection, each rodent was marked with a numbered ear tag (National Band & Tag Company^®^, New Port, RI, USA) at the base of its right ear and then returned to its capture site. Rodents recaptured within the same season were excluded from the statistical analyses.

### 2.4. Determination of Intrinsic Factors

Individuals with narrow anogenital distances were classified as females and those with wide distances as males. As HCC can vary according to the age of the individual [48], we only included adult rodents in the study. For this purpose, we considered females weighing more than 35 g and males weighing more than 40 g as adults [49]. Body condition was estimated by calculating a scalar mass index (SMI) as follows: SMI*_i_* = M*_i_ *[L_0_/L*_i_*]^bsma^, where M*_i_* and L*_i_* are the body mass and body length of the individual *i*, respectively. L_0_ is the arithmetic mean of the body length of the population, and bSMA is a scaling exponent derived from the standardized major-axis (SMA) regression of body mass on body length [50]. The parasitic load was calculated as the total number of ectoparasites collected from each individual.

### 2.5. Biochemical Validation of Enzyme Immunoassay (EIA)

To quantify hair GC concentrations, we opted to measure corticosterone, the primary GC present in rodents, using an EIA. The validation of the selected EIA (DetectX^®^ Corticosterone Enzyme Immunoassay kit K014-H5; Arbor Assays, Ann Arbor, MI, USA) involved a dilution and parallelism test [51]. In this process, we compared serial dilutions of a hair extract pool from the experimental samples with corticosterone standard concentrations provided by the manufacturer. The plotted curves represent the relationship between corticosterone concentration and the percentage of binding for the serial dilutions of the pooled hair extracts and the standards (Figure S1). By comparing both slopes with a Pearson correlation, we established a parallel displacement with the hormonal standard (r = 0.9988; *p* = 0.0012) and determined the adequate concentration factor required for the experimental samples.

### 2.6. Measurement of Hair Glucocorticoid Concentration

To measure HCC, we followed the protocols outlined by Mastromonaco et al. (2014) [52] and Stewart et al. (2020) [53]. Initially, two to three weeks after sampling, samples were subjected to a washing step to remove potential surface contaminants. Hair segments of 5 mm were placed in Eppendorf tubes, and 500 μL of methanol was added to each tube. After allowing the methanol to act for 10 s, the tube was vortexed, and the methanol was subsequently removed using a pipette.

For corticosterone extraction, 100% methanol was added to each hair sample at a ratio of 50 mg/mL. The samples were then placed on a plate shaker for 24 h. Subsequently, the tubes were centrifuged for 10 min at 2400 rpm to collect the supernatant, which was then stored at −20 °C. Before the immunoassay, the supernatant was evaporated to obtain dry extracts. These extracts were reconstituted by adding EIA buffer (DetectX^®^) at a 1:3 ratio of original extract to buffer volumes.

Finally, the quantification of corticosterone concentrations was performed using an enzyme immunoassay, utilizing a commercial kit (DetectX^®^ Corticosterone Enzyme Immunoassay kit K014-H5, Ann Arbor, MI, USA). The colorimetric absorbance of the reaction was then examined with a spectrophotometer at 450 nm. The optical density data obtained were inputted into the “MyAssays” platform to obtain the corticosterone concentration for each sample. This data analysis employs a Four Parameter Logistic (4PL) curve fit, following the protocol outlined in the enzyme immunoassay kit. All samples were run in duplicate, and only CVs < 10% were accepted.

### 2.7. Statistical Analyses

Statistical analyses were performed using R software (version 3.6.3). Given the normality of the corticosterone data (Figure S2), we evaluated Linear Mixed-effects Models (LMM) for HCC as the response variable and locality, seasonality, sex, body condition, and ectoparasite load as predictors, and sampling grid nested within locality as random effects. These analyses were run using the “lmer” function of the “lme4” package. We also run linear models, maintaining the variable response of the LMM and considering its fixed effects as predictor variables. Subsequently, the fit of both models was compared using the Akaike Information Criterion (AIC) with the “MuMIn” library. Based on the AIC comparison, we decided to proceed with linear models, which exhibited a better fit in all cases. The “autoplot” function was used to check the assumptions of the linear models (Figure S2), the significance of the effects of predictor variables on HCC was assessed using the “anova” function, and the magnitude of the effects was determined by evaluating the coefficients provided by the “summary” function.

## 3. Results

We collected hair from 199 adult individuals of *P. darwini* between the spring of 2021 and the summer of 2023, with 117 of them captured in the BFJNP and 82 in the rural locality of El Tangue, comprising 65 females and 134 males (Table 1). The mean value of HCC in the analyzed samples was 57.61 ng/g (s.d. = 20.82), 69.81 ng/g (s.d. = 23.44) for females and 51.69 ng/g (s.d. = 16.51) for males. Intra-assay and inter-assay coefficients of variation (%CV) for the corticosterone EIA were 6.26% and 7.5%, respectively.

**Figure 3:**
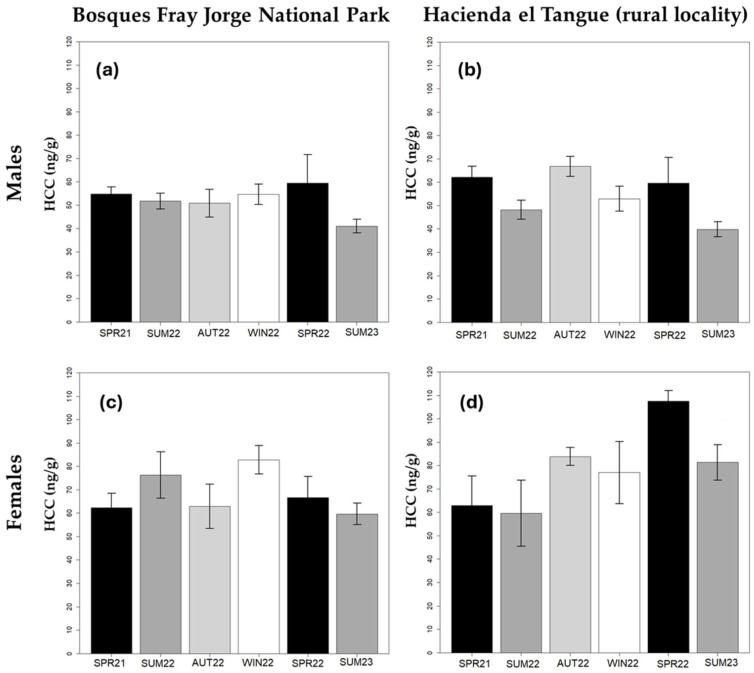
Seasonal variations in hair corticosterone concentration (HCC) of samplings obtained from *Phyllotis darwini* inhabiting the Bosques Fray Jorge National Park (BFJNP) and Hacienda el Tangue: (**a**) HCC in males from the BFJNP; (**b**) HCC in males from Hacienda el Tangue; (**c**) HCC of females from BFJNP; (**d**) HCC of females from Hacienda el Tangue. The graphs show the arithmetic mean and the standard error of each group.

Hair corticosterone concentration in *P. darwini* was significantly associated with sex (*p* < 0.001), with lower concentrations observed in males than in females (Table 2, Figure 3). Differences associated with the season were also evident, with lower concentrations found in summer 2022 and 2023 (Table 2). On the other hand, neither locality, body condition, nor ectoparasite load showed a significant effect on HCC. Given the substantial difference between sexes, we explored models for each gender separately. The specific model for males also showed seasonal fluctuations (Table 2), showing a lower hormone concentration in hair samples obtained in the summer of 2023 compared to the other seasons (Table 2 and Figure 3). Seasons between the spring of 2021 and the spring of 2022 did not show significant differences. On the other hand, males did not show differences in HCC between the anthropized locality of El Tangue and the protected wild area BFJNP (Figure 3). Additionally, no association was observed between these hormones in males and body condition or ectoparasite load. In the case of the model for females, none of the evaluated response variables showed a significant association with HCC.

## 4. Discussion

### 4.1. No Difference in Hair Corticosterone Levels between Anthropized Areas and Areas Protected from Human Disturbance

No differences were found in hair corticosterone concentrations between the groups of individuals inhabiting El Tangue and BFJNP. This finding is in disagreement with our prediction that rodents living in anthropized areas would exhibit higher concentrations of GC, as has been the trend in other mammals [9,10,32,54]. This could indicate that the stressors associated with anthropogenic activity in the area may not impose greater energy demands on these rodents, which could be related to the generalist habits of *P. darwini*. Other studies have attributed the lack of association between anthropization and GC concentrations to attenuations in the stress response to chronic stress [55]. Thus, physiological desensitization to stressors may have occurred due to downregulation of the hypothalamus–pituitary–adrenal axis, which can occur with prolonged and repeated exposure to certain stimuli, aiming to avoid the detrimental effects of chronic GC concentration elevations [56]. Alternatively, a habituation process might have occurred, meaning the animal could have learned to avoid incurring an energetically costly response (stress response) by not perceiving any benefit in responding to certain repeated stimuli [56,57].

On the other hand, although anthropization was expected to amplify the physiological stress faced by individuals living in drylands, it is possible that the energy demands imposed by adverse climatic conditions operate with greater magnitude and in a similar manner in both localities. In this way, the effect of anthropization on the total concentrations of hair corticosterone would be low and would not contribute to generating significant differences between El Tangue and the BFJNP.

### 4.2. Sex-Based Differences in Hair Corticosterone

Sex differences in GC concentrations are common among vertebrates [5,9,48] and have been attributed to differences in energy investment in social status or reproduction [11,12,13,58]. Higher concentrations of GC in females compared to males, as observed in this study, have frequently been reported in the literature [9,13,59]. This pattern is commonly reported and can be attributed to the fact that female *P. darwini* face higher energy demands than males during energetically costly periods such as lactation, gestation, or parental care [9,60,61]. During the reproductive period (October to February), females of this species can gestate two to three litters, giving birth on each occasion to five to six altricial pups [62], contributing to higher energy expenditure. However, the differences in HCC between sexes were evident throughout all sampling seasons and not only during the reproductive period. Additionally, studies in rodents have demonstrated differentiated stimulation of the HPA axis by sexual hormones, with the resulting stress response being potentiated by estradiol and inhibited by androgens [15,60,63], which could help to explain the differences found between sexes.

### 4.3. Seasonal Variation in Hair Corticosterone

The classical model of corticosterone incorporation into hair suggests that it occurs during its growth [64,65]. Although there are no descriptions of the hair growth cycle in *P. darwini*, after conducting hair shavings during seasonal fieldwork, we were able to confirm growth throughout all seasons, indicating that integration might take place continuously throughout the year. On the other hand, some studies suggest that corticosterone integration into hair could actively occur from the bloodstream, gradually increasing after a few hours of exposure to a stressor [66]. In this latter way, corticosterone would integrate throughout all seasons, regardless of whether the hair is in a growth phase or not. Studies on the hair growth dynamics in *P. darwini* would contribute to the understanding of HCC seasonal fluctuations. However, based on the aforementioned considerations, we assume that corticosterone integrates into the hair throughout the year.

Glucocorticoid levels tend to fluctuate seasonally in free-living wildlife [9,67]. This pattern is associated with the variation in energy requirements and resource availability throughout different seasons [4,67,68,69]. Seasonal variations in HCC in *P. darwini* were detected only in the overall and the male-specific models. This could be attributed to actual seasonal variations occurring only in males or, alternatively, to the limited sample size of females, which might have hindered the detection of any seasonal pattern.

Male rodents exhibited differences in corticosterone concentrations in hair samples obtained in the summer of 2023 compared to the preceding period, which spanned from spring 2021 to spring 2022. Specifically, a significant decrease in HCC was observed in this latter sampling season in both sampling sites (summer of 2023). A possible explanation for these results could be related to the water deficit conditions experienced in the area in 2021 and 2022. Although rainfall levels were not a variable analyzed in this study, the information available from meteorological stations indicates low precipitation throughout 2021, a condition that persisted until June 2022. From July to August 2022, rainfall events were more than 25 times higher compared to the monthly average between January 2021 and June 2022 (weather stations: Fray Jorge Quebrada, IEB-CEAZA, and Quebrada Seca, Ovalle, CEAZA [70]). The relationship between precipitation levels and population fluctuations of *P. darwini* in the semi-arid zone of Coquimbo is well-established [49,71,72]. Precipitation promotes primary production, enhancing food availability for this rodent in the subsequent months [71]. Moreover, male growth has been found to correlate positively with precipitation levels in this region [73]. Therefore, the observed decreases in hormone concentrations in hair samples collected in the summer of 2023 (January and February) are likely linked to the increased resources following the rainy season of 2022 that exceeded the previous years. This pattern may not have been noticeable in samples from the spring of 2022 (September and October) because HCC reflects cumulative concentrations in the period leading up to sampling [37,74], and the impact of rainfall on resources might not have been as pronounced during that time. During water scarcity periods, rodents likely faced challenges related to limited trophic resources and increased competition, both intra- and interspecific [2,49]. Given the favorable conditions following the rainy season [71], corticosterone concentrations could decrease.

Seasonal variations in GC concentrations associated with rainfall levels and higher GC concentrations in dry seasons have been observed previously in studies of free-living wildlife [68]. Although rainfall levels were not a variable analyzed in this study, given the background information provided, they are likely to be associated with GC concentrations in *P. darwini* and should be included in subsequent studies.

The sex-specific seasonal variations could be related to the differential stimulation of sexual hormones in the HPA axis during the reproductive period. The reproductive period of *P. darwini* typically extends between October and February [62]. During this period, males tend to increase their testosterone levels, affecting the activity of the HPA axis and decreasing GC concentrations, which has been evidenced in other rodent species [60]. This effect would make the decrease in corticosterone concentration in samples obtained in the summer of 2023 more evident in males.

### 4.4. No Association between Hair Corticosterone and Body Condition Nor Ectoparasite Load

Body condition has been linked to GC concentration in wildlife [48]. Frequently, poorer body condition is associated with higher concentrations of GC [18,19,59,75], explained by the need to mobilize energy from reserves to meet energy demands, a process regulated by these hormones [4,48]. Contrary to our predictions, we did not find a significant association between HCC and body condition in either the global model or the sex-specific models. The absence of a significant relationship between HCC and body condition has been observed in other studies involving small mammals, including black-tailed prairie dogs [76] and Alpine marmots [13]. Our results could be explained because the time measured by hair samples is too long in relation to the time that the body condition remains stable in *P. darwini* [73]. Therefore, a point measurement of the body condition on the day of hair sampling may not be representative of the studied period.

In relation to ectoparasites, their impact on energy reserves can be direct, as they consume nutrients from the host [22,77], or indirect, involving energy expenditures for the development and maintenance of the immune response [5,21]. Furthermore, ectoparasites may induce grooming behavior, diminishing the time allocated to rest and feeding, thereby contributing to increased energy expenditure [23,24,25]. Given the potential effects on energy demand, we predicted a positive association between ectoparasite loads and GC concentrations, aligning with trends observed in parasitic associations [27]. However, we did not observe a significant association between these variables. Our results may indicate that ectoparasitism in *P. darwini* might not entail significant energy expenditure, given the low parasite loads recorded for all taxonomic groups in both locations. The absence of a relationship between GC concentration and ectoparasite loads has been observed in other research [26], attributed to coevolutionary adaptation and a short duration of parasitic presence on the host [26,27,78]. In the case of coevolutionary adaptation, after a long coevolutionary history, parasites may not stimulate the anti-parasitic response of the host, and the host may experience less harmful effects from the interaction. Although the aforementioned could potentially explain our results, a comprehensive investigation would necessitate separate assessments of the relationship between GC concentrations and the load of each parasite species. Additionally, evaluating the phylogenetic proximity of *P. darwini* to the primary host of each parasite would provide further insights [26]. To determine whether the parasitic stage on the host contributes to our results, individual assessments for each parasite species and detailed information about their life cycle would be essential. However, given that HCC serves as an indicator of circulating GC concentrations over more extended periods compared to blood or fecal samples, detecting immediate effects on GC concentrations from parasite loads obtained on the day of sampling may be challenging.

### 4.5. Potential Limitations

Currently, there is controversy regarding the integration of corticosterone in hair due to acute stressors [66]. After challenging individuals with ACTH once, simulating exposure to an acute stressor, some species have shown no changes in HCC [79,80,81], while others have [66]. We cannot ignore the possibility that acute stress may affect HCC, leading to gradual increases in these hormones [66].

We opted for live capture traps (Sherman traps) in our study, taking bioethical considerations and the impact of lethal traps into account. Lethal traps could potentially impact corticosterone concentration to a lesser extent [32], but they also could introduce unwanted effects in a longitudinal study like ours. While live capture traps might induce acute stress in animals, all individuals in the study underwent the same capture method and experienced a similar stressor. Therefore, any potential increase in HCC due to capture should be observed similarly across all groups within the categories evaluated in this study (e.g., locality, season, sex). This should not significantly affect our interpretations, although it could potentially mask the effect of other factors, and a higher sample size may be necessary to observe these effects.

Another challenging limitation when working with wild rodents is the variation in capture times among individuals. Although this bias could be mitigated by reducing trap activation time, *P. darwini* exhibits nocturnal habits, making it impractical to restrict sampling to a few hours at night due to lighting conditions. Furthermore, limiting capture time would greatly reduce the number of animals captured in this study, which is particularly relevant in sites with low abundances. Despite these challenges, Colding et al. (2023) observed an increase in HCC in rodents after administering a corticosterone injection into the bloodstream, which was evident in their first measurement taken 3 h post-injection and remained stable until the subsequent measurement at 24 h post-injection. Therefore, we suggest that the difference in capture times between individuals likely had minimal effect on our HCC measurements, as captures mostly occurred overnight, and the traps were checked the following morning. Considering the aforementioned, differences in capture times could hinder the ability to distinguish the relationship between corticosterone and predictor variables, which could have weak effects on these hormone levels. However, they should not significantly affect variables with a stronger effect, such as seasonality and sex, as found in this study.

In enzyme-linked immunosorbent assays, the quantification of molecules depends on their binding to the assay antibody. It has been demonstrated that in some circumstances, when quantifying glucocorticoids in hair, other steroid hormones can adhere [82], which may vary their levels according to various factors, including sex and seasonality. Given this, it is beneficial to perform validations to determine the assay specificity. For the validation of our immunoassays, we present parallelism data and intra-assay and inter-assay coefficients of variation (%CV), and we do not perform other validations to determine specificity for corticosterone (e.g., stimulation with ACTH or HPLC immunogram). Despite this, the ELISA kit used in this study (DetectX^®^ Corticosterone Enzyme Immunoassay kit K014-H5; Arbor Assays, Ann Arbor, MI, USA) exhibits in most cases <1% cross-reactivity with other steroid hormones, including sexual hormones (progesterone, testosterone, estradiol) and their metabolites [83]. Therefore, this limitation would result in minimal bias in our results and would not interfere with our interpretation of seasonal and sex-related variations.

## 5. Conclusions

Our findings reveal no significant association between the degree of anthropogenic disturbance and HCC. This suggests that anthropogenic activity in the area might not impose greater energy demands on these rodents. Alternatively, the harsh environmental conditions of the semi-arid zone could have a more substantial impact on the energy demands of individuals, leading to increased GC concentrations both in anthropized and protected wild areas. While our study did not find a direct association between HCC and anthropization, we do not rule out the possibility that individuals’ stress levels could still be influenced. The lack of a discernible effect might be attributed to other unaccounted stressors, such as predation risk, which could potentially impact differently across localities. Moreover, the generalist habits of *P. darwini* might make our findings less applicable to more specialized rodent species. Conducting similar assessments in specialist species could provide valuable insights.

In addition, this study showed that the concentrations of GC in *P. darwini* inhabiting semi-arid environments seem to be primarily influenced by sex differences. This could be attributed to variations in energy investment in reproduction or distinct stimulation of sexual hormones in the HPA axis. Additionally, HCCs were influenced by factors that vary over time, including potential precipitation levels and primary productivity. While it is crucial to incorporate environmental and individual variables in assessments of GC concentrations, our results underscore the importance of integrating sex and seasonality in future studies involving *P. darwini*.

## Figures and Tables

**Table 1:** Number of sampled rodents, hair corticosterone concentration (HCC), body condition (scalar mass index SMI), and ectoparasite load (number of ectoparasites per individual) according to locality, sampling season, and sex. Ectoparasite load, HCC, and SMI columns show mean values and standard deviation.

Factor	BFJNP				El Tangue			
	N	HCC ng/g	SMI	Ectoparasitic Load	N	HCC ng/g	SMI	Ectoparasitic Load
*Sex*								
Male	81	51.6 (16.6)	52.5 (9.0)	2.9 (3.1)	53	51.8 (16.5)	53.5 (8.2)	3.9 (4.5)
Female	36	66.0 (20.0)	45.9 (8.2)	2.2 (2.5)	29	75.0 (27.1)	45.4 (6.7)	1.9 (2.0)
*Season*								
Spring 2021	42	56.7 (17.8)	49.4 (8.2)	2.0 (1.5)	14	62.5 (21.4)	46.9 (8.2)	4.1 (1.9)
Summer 2022	17	59.0 (18.8)	47.8 (6.4)	3.8 (3.3)	23	51.2 (22.5)	48.3 (6.0)	2.1 (4.1)
Autumn 2022	19	55.3 (22.4)	49.6 (9.0)	2.7 (3.2)	11	73.0 (13.0)	47.7 (8.2)	6.4 (5.2)
Winter 2022	10	63.1 (17.2)	49.9 (9.4)	4.9 (3.2)	9	63.6 (22.2)	52.6 (9.7)	4.6 (5.1)
Spring 2022	8	62.2 (22.6)	48.2 (8.4)	1.2 (1.8)	5	78.7 (29.8)	54.2 (10.6)	5.0 (1.9)
Summer 2023	21	47.2 (14.3)	56.8 (11.5)	3.3 (4.2)	20	54.4 (25.1)	55.4 (8.7)	1.2 (1.6)

**Table 2:** Final global (n = 199) and male (n = 134) models of factors predicting HCC of the Darwin’s leaf-eared mouse *P. darwini* in a semi-arid zone of northern Chile. Categories of comparison for each categorical variable are indicated in parentheses within the table.

Variable	Estimate	Std. Error	t Value	Pr (>|t|)
*Global Model*				
(Intercept)	62.417	3.462	18.031	<0.001 *
Sex (Male)	−17.788	2.832	−6.282	<0.001 *
Season (Summer 2023)				
Spring 2021	8.154	3.840	2.123	0.035 *
Summer 2022	4.962	4.155	1.194	0.234
Autumn 2022	10.644	4.487	2.372	0.019 *
Winter 2022	12.165	5.183	2.347	0.020 *
Spring 2022	17.072	5.945	2.872	0.005 *
*Male Model*				
(Intercept)	40.432	2.989	13.528	<0.001 *
Season (Summer 2023)				
Spring 2021	15.838	3.888	4.073	<0.001 *
Summer 2022	9.231	4.153	2.223	0.028 *
Autumn 2022	16.286	4.650	3.502	0.001 *
Winter 2022	13.501	5.388	2.506	0.013 *
Spring 2022	19.031	6.251	3.044	0.003 *

* = Statistically significant.

## Data Availability

Data generated in this study are openly available.

## Supplementary Materials

The following supporting information can be downloaded at: https://www.mdpi.com/article/10.3390/ani14091260/s1, Figure S1: Parallelism for serial dilutions of pooled hair extracts against the corticosterone standard curve; Figure S2: Distribution of hair corticosterone concentration (HCC) data in samples obtained from *Phyllotis darwini* and diagnostic graphs to check the assumptions of the linear models.
