# Supplementary material for: Seasonal Hair Glucocorticoid Fluctuations in Wild Mice (Phyllotis darwini) within a Semi-Arid Landscape in North-Central Chile
Source: Animals (Basel). 2024 Apr 23;14(9):1260. doi: 10.3390/ani14091260 (PMC11083726; doi:10.3390/ani14091260)

Figure S1. Parallelism curves for pooled hair extract from *Phyllotis darwini*. Serial dilutions of pooled hair extract are shown in red, and standard curve is shown in black with each concentration indicated with a diamond.

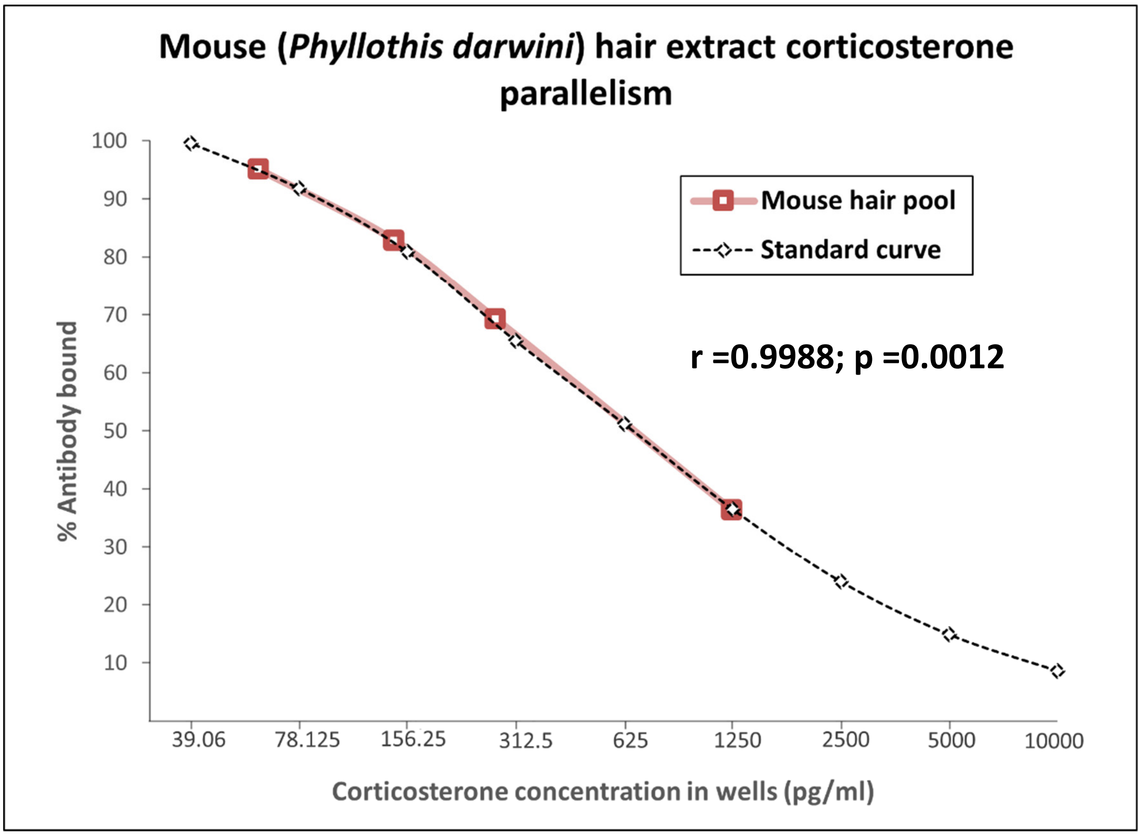

Supplement: Supplementary file 1 [file animals-14-01260-s001.zip › Figure S1.pdf]
