# Supplementary material for: Seasonal Hair Glucocorticoid Fluctuations in Wild Mice (Phyllotis darwini) within a Semi-Arid Landscape in North-Central Chile
Source: Animals (Basel). 2024 Apr 23;14(9):1260. doi: 10.3390/ani14091260 (PMC11083726; doi:10.3390/ani14091260)

Figure S2. Distribution of hair corticosterone concentration (HCC) data in samples obtained from *Phyllotis darwini* and diagnostic graphs to check the assumptions of the linear models. (a) Frequency histogram for HCC of all sampled individuals, (b) of males and (c) of females, (d) plot of residuals vs fitted values of the final global model lm(HCC~ sex+season), (e) of the final males model lm(HCC~ season) and (f) of the evaluated females model that included all predictors of the study lm(HCC~locality+season+body condition (scalar mass index)+ectoparasite load). Quantile-quantile plot which draws the correlation between the normal distribution and a given sample for the final global model (g), for the final male model (h) and for the evaluated female model (i). Each frequency histogram shows the statistical test used to evaluate normality and its results.

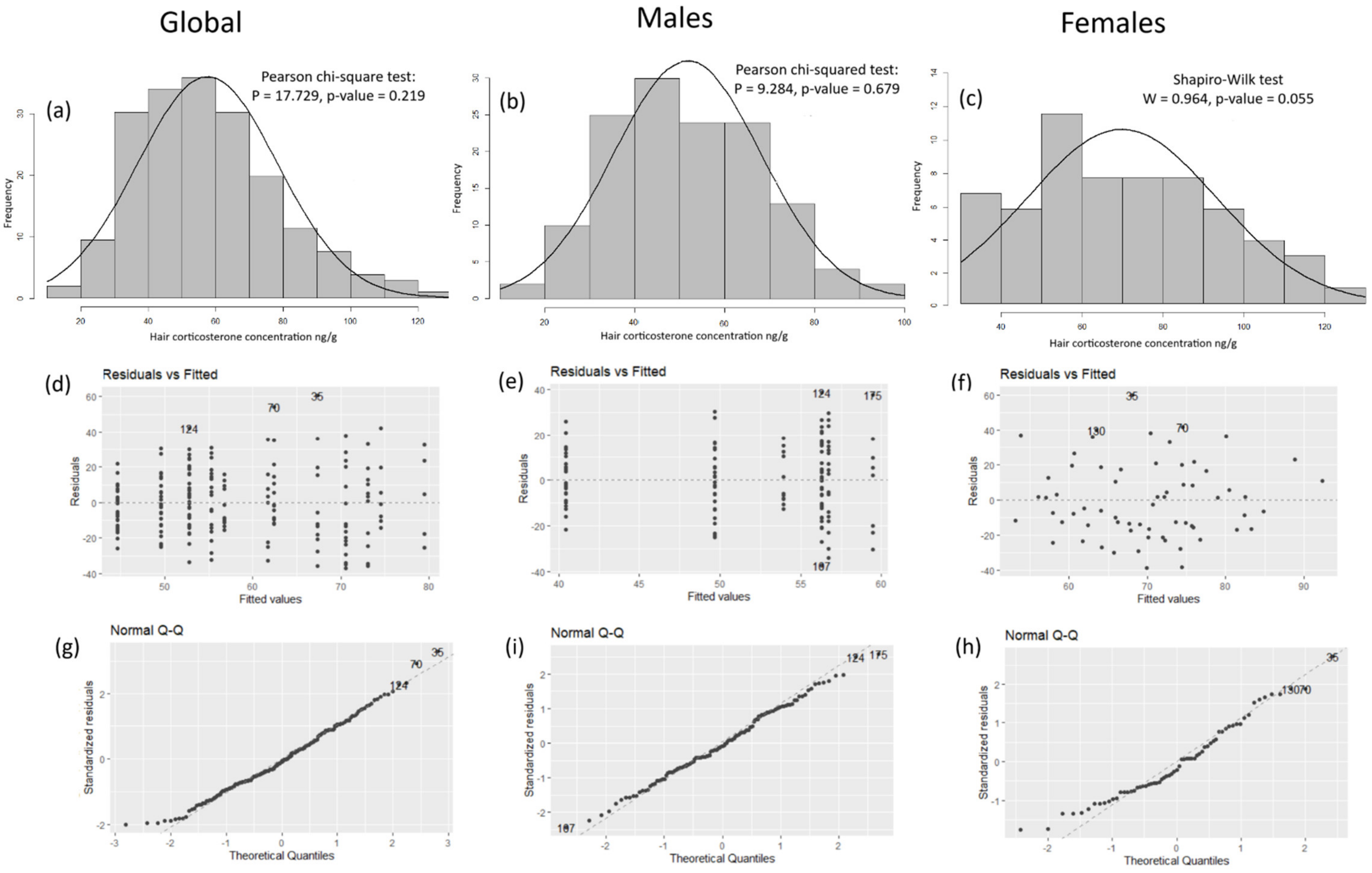

Supplement: Supplementary file 1 [file animals-14-01260-s001.zip › Figure S2.pdf]
